# Review of Facklamia Species Involvement in Human Infection and Urological Disease

**DOI:** 10.3390/microorganisms14061269

**Published:** 2026-06-04

**Authors:** Malika Saxena, Brian I. Choi, Alan J. Wolfe

**Affiliations:** Department of Microbiology and Immunology, Stritch School of Medicine, Maywood, IL 60153, USA; msaxena1@luc.edu (M.S.); bchoi2@luc.edu (B.I.C.)

**Keywords:** Aerococcoceae, *Facklamia*, microbiome, urinary tract infection, urogynecology, urobiome, uropathogen

## Abstract

*Facklamia* species are Gram-positive, catalase-negative cocci within the family Aerococcaceae. Historically misidentified as streptococci or enterococci due to phenotypic similarity and conventional biochemical testing limitations, their clinical significance remains incompletely defined. This narrative review synthesizes the current literature on the microbiology, epidemiology, clinical manifestations, diagnostic challenges, and antimicrobial susceptibilities of *Facklamia* infections, with emphasis on urogynecologic relevance. A structured literature search identified peer-reviewed reports of microbiologically confirmed human infection published through December 2025. These reports indicate global distribution and a broad clinical spectrum. Invasive infections were reported most often in older adults and individuals with structural abnormalities, chronic comorbidities, or recent surgical interventions; however, cases in otherwise healthy patients are described. Emerging microbiome data suggest that *F. hominis* may be enriched in adult females with lower urinary tract symptoms, supporting the possibility that the female urogenital tract functions as a potential reservoir and site of pathogenic activity. Antimicrobial susceptibility patterns vary, with documented resistance to penicillin, macrolides, clindamycin, and tetracyclines, and susceptibility to cephalosporins, vancomycin, and linezolid. Overall, *Facklamia* species should be recognized as underdiagnosed opportunistic pathogens with both invasive and urogynecologic relevance. Increased awareness, improved diagnostic identification, and systematic susceptibility profiling are needed to clarify their pathogenic role and guide appropriate antimicrobial therapy.

## 1. Introduction

Urogynecologic conditions are highly prevalent among adult females, encompassing a wide range of pelvic floor disorders with significant implications for quality of life and overall well-being [1]. Among the most common urogynecologic conditions are urinary tract infections (UTIs), which are estimated to affect 150 million people worldwide each year and account for substantial healthcare costs. UTIs are particularly prevalent in females, with up to 50% experiencing at least one episode over their lifetime and a significant proportion suffering from recurrent infections. Pelvic organ prolapse and urinary incontinence, which can contribute to frequent UTIs, are also believed to affect between 30% and 50% of females over the age of 50 [2].

The high prevalence of these conditions underscores the need for a comprehensive understanding of their causative agents and the potential role of emerging uropathogens. While the traditional causative agents of UTIs, such as *Escherichia coli*, have been extensively studied, recent research increasingly suggests that there may be a role for other, emerging uropathogens in the development and recurrence of these conditions [3]. These novel microbes, including a diverse array of Gram-negative and Gram-positive bacteria, as well as some fungi, present unique challenges in terms of accurate diagnosis and effective treatment, as they may exhibit resistance to traditional antimicrobial therapies. Furthermore, underlying host factors, such as age, diabetes, spinal cord injury, or catheterization, also can contribute to the development and recurrence of UTI, complicating the clinical management of these conditions [4].

Several of these emerging uropathogens are members of the family Aerococcaceae, a group of lactic-acid-producing, Gram-positive, catalase- and oxidase-negative, microaerophilic, nonmotile bacteria that have been increasingly implicated in urogynecologic conditions [5]. Among the most clinically recognized members of the Aerococcaceae family are the genera *Aerococcus*, *Globicatella*, *and Facklamia*. Historically, these genera have been underdiagnosed due to phenotypic similarity to viridans group streptococci and limitations of conventional laboratory identification systems [6,7,8].

*Aerococcus* species—particularly *A. sanguinicola* and members of the *A. urinae* complex—are routinely isolated from urine and have emerged as notable causes of UTIs, urosepsis, and infective endocarditis, especially in older adults and individuals with underlying urologic abnormalities [6].

*Globicatella* species, though less frequently reported, have been isolated from urine, blood, and central nervous system infections and are increasingly recognized in invasive disease [9].

Members of the genus *Facklamia* have been isolated numerous times from urine samples obtained from adult females with and without symptoms of recurrent UTI [10]. *Facklamia* species also have been isolated in seemingly unrelated conditions, suggesting a role as an opportunistic pathogen [11]. In this review, we present a general history of the genus *Facklamia*, clinically relevant cases described in the literature thus far, and an analysis of the role of *Facklamia* in urogynecologic conditions.

*Facklamia* species are Gram-positive, α-hemolytic, catalase-negative, oxidase-negative, facultative anaerobic cocci. They have been identified in the literature primarily in cases of urosepsis, skin abscesses, peritonitis, meningitis, endocarditis, chorioamnionitis, and pyelonephritis [11]. Even though Matrix-Assisted Laser Desorption/Ionization Time-of-Flight (MALDI-TOF) mass spectrometry has improved recognition of *Facklamia*, the genus remains elusive due to phenotypic similarity to streptococci, limited representation in older identification databases, and entrenched assumptions that can lead to misclassification or dismissal as a contaminant [12]. Although *Facklamia* species have been implicated in invasive infections, the virulence of this microbe has not been fully determined. The true extent of morbidity and mortality is further convoluted by the relative scarcity of reported cases and by the co-occurrence of *Facklamia* with other bacterial species, often in patients with significant comorbidities. In addition to blood cultures, *Facklamia* has been isolated from urine, vaginal specimens, cerebrospinal fluid, bone, skin, gallbladder, and breast [13].

The genus *Facklamia* was first proposed in 1997 to accommodate a group of Gram-positive, catalase-negative, coccoid-ovoid bacteria that were previously classified as aerococci or streptococci. The type species, *F. hominis*, was initially isolated from human clinical specimens and differentiated from the closely related genus *Globicatella*, based on various phenotypic and genotypic characteristics [13].

*F. hominis* is distinguished from *Globicatella* species by several key features. *F. hominis* cells are generally smaller in size, ranging from 0. 5 to 1 μm in diameter, compared to the larger 1–2 μm cells of *Globicatella* species. While *Globicatella* species are typically oxidase-positive, *F. hominis* is generally oxidase-negative. Furthermore, *F. hominis* can be differentiated from *Globicatella* species by its inability to grow at 45 °C and, in contrast to *Globicatella*, its lack of acid production from mannitol. *Facklamia* also differs due to its type A4α murein, unlike the type Ala murein that *Globicatella* possesses [14].

The other three known *Facklamia* species that have been isolated in humans—*F. ignava*, *F. sourekii*, and *F. languida*—can be distinguished from *F. hominis* through a combination of phenotypic and genotypic characteristics. *F. ignava*, isolated from vaginal swabs, can be differentiated by its ability to produce acid from sucrose, raffinose, or trehalose, which *F. hominis* cannot do. *F. sourekii* differs from *F. hominis* in its ability to hydrolyze esculin and produce acid from mannose. Finally, *F. languida*, isolated from a human clinical source, is unique among *Facklamia* species in its inability to produce acid from glucose, lactose, or maltose [14].

The diversity within the *Facklamia* genus highlights the challenges in accurately identifying and differentiating these bacteria, which can be easily confused with other catalase-negative, Gram-positive cocci, such as *Enterococcus* and *Streptococcus*. In the past, accurate identification typically required biochemical testing, sometimes combined with 16S rRNA gene sequencing analysis. Today, however, MALDI-TOF plays a more substantial role.

The purpose of this review is to comprehensively characterize the genus *Facklamia* within the context of human disease, with an emphasis on its role in urogynecologic conditions. Specifically, we aim to (1) synthesize all published cases of microbiologically confirmed *Facklamia* infection to define its clinical spectrum and invasive potential; (2) evaluate reported diagnostic methodologies and highlight challenges contributing to historical under-recognition; (3) summarize available antimicrobial susceptibility data to inform therapeutic decision-making; and (4) contextualize emerging microbiome evidence suggesting a potential association between *F. hominis* and female lower urinary tract symptoms. By integrating invasive case reports with contemporary urogenital microbiome findings, this review seeks to clarify the clinical significance of *Facklamia* species and identify key gaps requiring further investigation.

## 2. Materials and Methods

A structured literature review was conducted to identify published cases of *Facklamia* infections across clinical contexts. A comprehensive search of PubMed/MEDLINE, Embase, and Google Scholar was performed using combinations of the terms “Facklamia,” “Facklamia infection,” “Facklamia bacteremia,” “Facklamia urinary tract infection,” “Facklamia endocarditis,” and “Facklamia pregnancy.” Articles published in English from database inception through 12/25 were considered.

All peer-reviewed case reports, case series, and observational studies describing microbiologically confirmed *Facklamia* infection in humans were eligible for inclusion. Studies were excluded if species identification was not confirmed, if the microbe was reported without associated clinical infection, or if insufficient clinical data were provided. Non-English articles without accessible translations were excluded. Data extracted included patient demographics, geographic location, infection site, microbiologic identification method (including MALDI-TOF or sequencing, when reported), antimicrobial susceptibilities, treatment regimen, and clinical outcomes. Given the heterogeneity and predominance of case-level data, findings were synthesized descriptively without meta-analysis.

## 3. Results

### 3.1. Literature Search

As described above, a structured literature search was conducted to identify peer-reviewed reports of microbiologically confirmed human *Facklamia* infections published through December 2025. After removal of duplicates and screening for relevance, eligible articles consisted primarily of case reports and small case series. From each included study, data were systematically extracted on patient demographics, comorbidities and predisposing factors, species identified, site of isolation, clinical presentation, diagnostic methodology, antimicrobial therapy, and susceptibility patterns when available. These data were synthesized into a comprehensive summary table to characterize the epidemiology, clinical spectrum, and treatment patterns associated with *Facklamia* infections (Table 1).

### 3.2. Diagnosis

In cases of invasive disease, isolation of Facklamia most commonly occurred from blood cultures, although members of this genus also have been recovered from abscess material, peritoneal fluid, cerebrospinal fluid, urine, wound cultures, and prosthetic interface tissue. In the cases summarized in the attached table (*n* = 33 with reported specimen source), blood was the most frequent source of isolation (15/33, ~45%), followed by abscess or localized soft tissue collections (3/33, ~9%). Other specimen sources included peritoneal fluid (1/33, ~3%), cerebrospinal fluid (2/33, ~6%), urine (1/33, ~3%), wound secretion cultures (1/33, ~3%), urethral exudate (1/33, ~3%), cyst fluid (1/33, ~3%), placental or obstetric tissue specimens (1/33, ~3%), gallbladder (1/33, ~3%), and cases with no clearly specified source (6/33, ~18%) (Table 1).

Based on the cases summarized in the attached table (*n* = 33 with identifiable methods reported), MALDI-TOF was the most frequently used modality, accounting for 11/33 cases (~33%), with the Vitek2 system used in 4 of these 11. 16S rRNA gene sequencing served as the primary identification method in 11/33 cases (~33%) while PCR was reported in 2/33 (~6%). Conventional biochemical systems, such as API Rapid ID32 and Strep/API ZYM, were used in 2/33 cases (~6%). In 2 cases (~6%), the identification method was not specified (Table 1). These data illustrate a temporal shift from biochemical phenotyping toward proteomic (i.e., MALDI-TOF) or DNA-based (i.e., PCR and 16S rRNA gene sequencing) techniques. The broader availability of these advanced diagnostic tools likely explains the increasing number of reported cases and supports the conclusion that infections were historically underrecognized due to limitations of earlier identification systems. Polymicrobial infection is described in several cases, particularly in intra-abdominal or soft tissue infections, which may complicate interpretation of culture results and obscure the microbe’s clinical significance. Notably, polymicrobial growth is reported in select peritoneal and central nervous system infections within the cohort, underscoring the diagnostic challenge of determining whether *Facklamia* represents a primary pathogen or a co-isolated microbe in complex infectious presentations (Table 1).

### 3.3. Therapy

Across the cases summarized in the attached table (*n* = 33), third-generation cephalosporins were the most frequently administered agents (6/33, 18%), followed by vancomycin (4/33, 12%), gentamicin (4/33, 12%), and amoxicillin-clavulanate (3/33, 9%), with overall favorable outcomes in the majority of treated patients; however, documented penicillin resistance (reported in at least 3 cases) and additional resistance to macrolides, clindamycin, tetracycline, and trimethoprim–sulfamethoxazole (TMP-SMX) in select isolates reinforce the concept that empiric β-lactam monotherapy—particularly penicillin alone—may be inadequate without susceptibility-guided therapy (Table 1).

## 4. Discussion

Invasive *Facklamia* infections most commonly manifest as endocarditis and bacteremia. Structural cardiac abnormalities, including rheumatic valvular disease and other preexisting cardiac conditions, appear to be prominent risk factors, particularly among patients who develop endocarditis. Advanced age, diabetes mellitus, obesity, malignancy, cirrhosis, and other chronic comorbidities are frequently documented in reported cases. The presence of prosthetic material, such as joint prostheses, as well as recent surgical or urologic procedures, also appears to increase susceptibility, likely by disrupting normal epithelial barriers. While the majority of cases occurred in medically complex or immunocompromised individuals, occasional infections are described in younger or otherwise healthy patients, indicating that pathogenicity is not exclusively confined to high-risk populations.

Beyond invasive disease, emerging microbiologic data suggest that *F. hominis* may have a more substantial role in female lower urinary tract pathology than previously appreciated. In a cohort of 1004 women, *F. hominis* was identified in 4.07% of all samples but was disproportionately represented in patients with urinary (UUI) incontinence, where detection reached 12.25%, compared with only 1.42% among controls. Prevalence was lower in uncomplicated urinary tract infection (1.32%) and stress urinary incontinence (2.00%), and absent in interstitial cystitis/painful bladder syndrome cohorts [30]. This enrichment in UUI suggests that *F. hominis* may contribute to lower urinary tract symptomatology rather than serving solely as an incidental colonizer. Together with prior reports of obstetric and genitourinary infections, these findings support the hypothesis that the female urogenital tract represents both a reservoir and a potential site of pathogenic activity.

The clinical presentation of invasive *Facklamia* infection appears to be nonspecific and varies according to the site involved. Endocarditis often presents with typical features of subacute bacterial endocarditis, whereas bacteremia may occur with or without a clear primary focus. Additional manifestations reported in the literature include urosepsis, peritonitis, meningitis, deep soft tissue abscesses, pneumonia progressing to ARDS, and prosthetic joint infection. Because these presentations overlap with more common pathogens, clinical suspicion is rarely directed toward *Facklamia* species at initial evaluation.

Furthermore, diagnosis remains challenging. Earlier identification relied on conventional biochemical systems, such as the API tests, which occasionally resulted in misclassification as streptococci or enterococci. However, the increasing implementation of MALDI-TOF mass spectrometry has significantly improved diagnostic accuracy, with 16S rRNA gene sequencing used for confirmation in ambiguous cases.

An additional observation is the apparent difference in antimicrobial susceptibility patterns based on identification methodology. Earlier reports relying on biochemical or enzymatic identification methods demonstrated relatively uniform susceptibility profiles, most commonly showing penicillin susceptibility with resistance to clindamycin and cefotaxime. In contrast, cases identified using MALDI-TOF or molecular techniques revealed broader and more heterogeneous resistance patterns, especially among *Facklamia hominis* isolates, including resistance to penicillin, macrolides, tetracyclines, and other agents. This discrepancy may reflect the limited discriminatory capacity of earlier biochemical methods, resulting in species misidentification and an artificially uniform resistance profile. Alternatively, improved species-level resolution afforded by MALDI-TOF and molecular approaches may better capture intrinsic interspecies variability in susceptibility patterns. Temporal changes in antimicrobial exposure and selective pressures may also contribute to evolving resistance profiles. These findings underscore the importance of modern identification techniques for accurate species determination and antimicrobial susceptibility interpretation in *Facklamia* infections.

Therapeutic strategies have varied, largely reflecting empiric management for suspected enterococcal or streptococcal infection. Third-generation cephalosporins, vancomycin, amoxicillin-clavulanate, and gentamicin have been most frequently administered, particularly in cases of endocarditis, where combination therapy was common. Clinical outcomes were generally favorable with targeted antimicrobial therapy, although severe and fatal cases have been reported. Notably, penicillin resistance was the most consistently documented resistance pattern across cases. Additional resistance to macrolides, clindamycin, tetracycline, and trimethoprim–sulfamethoxazole has been observed in select isolates. In contrast, susceptibility to ceftriaxone, vancomycin, and linezolid was frequently retained.

## 5. Conclusions

To our knowledge, this synthesis represents one of the most comprehensive narrative summaries integrating invasive case reports with emerging microbiome data regarding female urogenital involvement. However, the literature remains limited to case reports and small case series, introducing reporting bias, incomplete susceptibility data, and heterogeneity in diagnostic methodology. The observed association between *F. hominis* and UUI is observational and remains insufficient to establish causality.

In conclusion, *Facklamia* species should be recognized as opportunistic yet potentially invasive pathogens with dual clinical relevance: systemic infection in patients with structural or chronic comorbidities and a possible contributory role in female lower urinary tract dysfunction. Future studies should include prospective clinical cohorts evaluating the prevalence of *F. hominis* in adult females with UUI compared to asymptomatic controls, mechanistic investigations into urothelial inflammatory pathways, and standardized antimicrobial susceptibility profiling to better define resistance patterns. Importantly, while *F. hominis* accounts for a large plurality of reported cases in this synthesis (*n* = 14), other species such as *F. ignava* (*n* = 7), *F. languida* (*n* = 8), and *F. soureki* (*n* = 3) also have been implicated in invasive infections—including bacteremia, endocarditis, ARDS, and pneumonia—yet remain poorly characterized microbiologically and clinically. Dedicated genomic, virulence, and resistance studies of these less frequently reported species are warranted to clarify whether pathogenic potential and antimicrobial susceptibility differ across the genus and to determine whether current empiric treatment strategies are universally appropriate.

## Figures and Tables

**Table 1:** Reports of *Facklamia* Infection.

l	Age, Sex ^1^	Underlying Condition ^2^	Infection ^3^	Sample ^4^	Identification Method ^5^	Antibiotic Treatment	Co-Isolates	Resistance Profile ^6^	*Facklamia* Species
1998, Canada [14]	1, F	Unknown	NR	Blood	16S rRNA	NR	NR		*F. ignava*
1998, Canada [14]	76, F	Unknown	NR	Blood	16S rRNA	NR	NR		*F. ignava*
1998, Canada [14]	48, F	Unknown	NR	Blood	16S rRNA	NR	NR		*F. ignava*
1998, USA [14]	82, F	Long-term nursing home	Pneumonia	Blood	16S rRNA	NR	NR		*F. ignava*
1998, USA [15]	86, F	Unknown	Septicemia	Blood	16S rRNA	NR	NR		*F. ignava*
2000, USA [15]	6, F	Unknown	Bacteremia	Blood	Biochemical/ enzymatic ID	NR	NR	S: Penicillin; R: Clindamycin, Cefotaxime	*F. languida*
2000, USA [15]	62, F	Unknown	Cardiac arrest	Blood	Biochemical/ enzymatic ID	NR	NR	S: Penicillin; R: Clindamycin, Cefotaxime	*F. languida*
2000, Canada [15]	74, F	Unknown	UTI	Blood	Biochemical/ enzymatic ID	NR	NR	S: Penicillin; R: Clindamycin, Cefotaxime	*F. languida*
2000, Canada [15]	83, F	Unknown	NR	Blood	Biochemical/ enzymatic ID	NR	NR		*F. ignava*
2000, Canada [15]	86, F	Unknown	NR	Blood	Biochemical/ enzymatic ID	NR	NR	S: Penicillin; R: Clindamycin, Cefotaxime	*F. languida*
2000, USA [15]	NR, F	Unknown	NR	Gallbladder	Biochemical/ enzymatic ID	NR	NR	S: Penicillin; R: Clindamycin, Cefotaxime	*F. languida*
2000, Sweden [15]	40, F	Unknown	NR	CSF culture	Biochemical/ enzymatic ID	NR	NR	S: Penicillin; R: Clindamycin, Cefotaxime	*F. languida*
2000, Sweden [15]	NR	Unknown	NR	Blood	Biochemical/ enzymatic ID	NR	NR	S: Penicillin	*F. soureki*
2000, Canada [15]	NR	Unknown	NR	Blood	Biochemical/ enzymatic ID	NR	NR	S: Penicillin	*F. soureki*
2000, France [15]	NR	Unknown	NR	NA	Biochemical/ enzymatic ID	NR	NR	S: Penicillin	*F. soureki*
2004, UK [16]	34, F	Pregnancy	Chorioamnionitis, puerperal bacteremia, maternal sepsis	Placental tissue swabs, placental tissue, gastric aspirate of the newborn infant	NR	Amoxicillin and clavulanic acid, Metronidazole	Group B streptococci and Coagulase-negative staphylococci	S: Penicillin, Ampicillin; R: Erythromycin, Tetracycline	*F. hominis*
2010, UK [17]	NR, NR	Cardioembolic stroke	Infective endocarditis	Blood culture	PCR	Gentamicin and Vancomycin	NR	R: Penicillin	*F. hominis*
2012, India [18]	35, M	Rheumatic mitral valve stenosis	Endocarditis	Blood culture	MALDI-TOF	Ceftriaxone, Gentamicin	NR	R: Penicillin	*F. hominis*
2014, Spain [19]	81, F	Obesity	Prosthetic joint infection	Peri-prosthetic femoral Prosthetic tissue, femoral interface membrane, acetabular interface membrane	MALDI-TOF	Amoxicillin, Ceftriaxone	NR	R: Erythromycin, Tetracycline	*F. hominis*
2015, France [20]	40, F	None	Scapular abscess		MALDI-TOF	Pristinamycine	16S rRNA PCR identified as *Enterococcus faecalis*	R: Clindamycin	*F. hominis*
2015, USA [7]	41, F	Recurrent sinusitis	Meningitis	CSF culture	MALDI-TOF	Ceftriaxone	*Streptococcus pneumoniae* (in blood + CSF)		*F. hominis*
2017, Germany [21]	63, M	Pulmonary metastases of melanoma	Thoracic abscess following epidural catheter	Abscess culture	PCR	Clindamycin	NR		*F. hominis*
2017, USA [11]	64, M	IV drug use	Endocarditis	Blood	16S rRNA	Gentamicin, Vancomycin	MALDI-TOF misidentified it as *Streptococcus viridans*		*F. ignava*
2017, USA [11]	36, M	Depression	ARDS	Blood	16S rRNA	Vancomycin	MALDI-TOF misidentified it as *Abiotrophia defectiva*		*F. languida*
2017, USA [11]	59, F	Diabetes, CKD, NAFLD	Cardiorespiratory failure	Blood	16S rRNA	Linezolid and Ceftriaxone (targeted towards *Staph aureus*)	*Staphylococcus aureus*, *Peptostreptococcus anaerobius*		*F. languida*
2019, Spain [22]	9, M	Phimosis + balanopreputial adhesions	Balanoposthitis	Urethral exudate culture (penile)	MALDI-TOF	Amoxicillin and clavulanic acid	NR	S: Cefotaxime, Levoflox, Linezolid, Ampicillin, Vancomycin; R: Azithromycin	*F. hominis*
2019, Korea [23]	67, M	Hepatitis B virus-cirrhosis	Epidermal cyst	Wound secretion culture	MALDI-TOF	Amoxicillin and clavulanic acid	NR		*F. hominis*
2020, Switzerland [24]	82, M	Benign prostatic hyperplasia + transurethral prostate resection	Urosepsis	Blood	MALDI-TOF	Ceftriaxone Cotrimoxazole	NR		*F. hominis*
2020, France [25]	57, F	Uterine myomatosis, hysterectomy, salpingooophorectomy, obesity	Peritonitis	Peritoneal culture	MALDI-TOF	Cefotaxime, Metronidazole, Amoxicillin	*E. coli*, *K. pneumoniae*, *Bacteroides fragilis*, *Enterococcus faecalis*, and multiple colonies of *F. hominis*		*F. hominis*
2021, USA [26]	64, F	Type 2 diabetes mellitus and coronary artery disease, Hurley stage III Hidradenitis Suppurativa	Hidradenitis suppurativa	Axillary abscess culture	MALDI-TOF	Moxifloxacin, Vancomycin, Amoxicillin	NR	R: TMP-SMX	*F. hominis*
2021, Mexico [27]	7, F	Congenital lymphangioma	Pyelonephritis	Urine culture	MALDI-TOF	Gentamicin	NR	R: Vancomycin, Gentamycin, Tetracycline, Linezolid	*F. hominis*
2024, Australia [28]	62, F	Bilateral breast cancer, left upper extremity lymphedema	Sebaceous cyst of breast	Cyst culture	NR	Flucloxacillin	NR	NR	*F. hominis*
2025, India [29]	55, M	Grade IV filarial lymphedema	No active infection (skin microbiome colonization)	Leg skin swab	16S rRNA	NR	Mixed skin microbiota (not individually specified)	NR	*Facklamia* spp. (species not resolved)
2025, India [29]	58, M	Grade III filarial lymphedema; hypertension	No active infection (skin microbiome colonization)	Leg skin swab	16S rRNA	NR	Mixed skin microbiota (not individually specified)	NR	*Facklamia* spp. (species not resolved)
2025, India [29]	50, M	Grade IV filarial lymphedema	No active infection (skin microbiome colonization)	Leg skin swab	16S rRNA	NR	Mixed skin microbiota (not individually specified)	NR	*Facklamia* spp. (species not resolved)

^1^ F, female; M, male; NR, not reported. ^2^ CKD; chronic kidney disease; IV, intravenous; NAFLD, nonalcoholic fatty acid liver disease; ^3^ ARDS, acute respiratory distress syndrome; UTI, urinary tract infection; ^4^ CSF, cerebrospinal fluid; ^5^ MALDI-TOF, matrix-assisted laser desorption/ionization time-of-flight; PCR, polymerase chain reaction; ^6^ R, resistant; S, sensitive; TMP-SMX, trimethoprim–sulfamethoxazole.

## Data Availability

The original contributions presented in this study are included in the article. Further inquiries can be directed to the corresponding author.

## Abbreviations

The following abbreviations are used in this manuscript:

MALDI-TOF	Matrix-assisted laser desorption/ionization time-of-flight
PCR	Polymerase chain reaction
TMP/SMX	Trimethoprim–sulfamethoxazole
UTI	Urinary tract infection
UUI	Urgency urinary incontinence

## References

[1] Peinado-Molina R.A., Hernández-Martínez A., Martínez-Vázquez S., Rodríguez-Almagro J., Martínez-Galiano J.M. (2023). Pelvic floor dysfunction: Prevalence and associated factors. BMC Public Health.

[2] Czajkowski K., Broś-Konopielko M., Teliga-Czajkowska J. (2021). Urinary tract infection in women. Prz. Menopauzalny.

[3] Whelan S., Lucey B., Finn K. (2023). Uropathogenic *Escherichia coli* (UPEC)-associated urinary tract infections: The molecular basis for challenges to effective treatment. Microorganisms.

[4] Hibbing M.E., Conover M.S., Hultgren S.J. (2016). The unexplored relationship between urinary tract infections and the autonomic nervous system. Auton. Neurosci..

[5] Maezawa Y., Nagasaki K. (2024). *Aerococcus urinae*: An emerging, gram-positive pathogen causing urinary tract infection. Am. J. Med..

[6] Rasmussen M. (2013). Aerococci and aerococcal infections. J. Infect..

[7] Parvataneni K.C., Iyer S., Khatib R., Saravolatz L.D. (2015). Facklamia Species and *Streptococcus pneumoniae* Meningitis: A Case Report and Review of the Literature. Open Forum Infect. Dis..

[8] Miller A.O., Buckwalter S.P., Henry M.W., Wu F., Maloney K.F., Abraham B.K., Hartman B.J., Brause B.D., Whittier S., Walsh T.J. (2017). *Globicatella sanguinis* osteomyelitis and bacteremia: Review of an emerging human pathogen with an expanding spectrum of disease. Open Forum Infect. Dis..

[9] Rîpă C.V., Cobzaru R.G., Iancu L.S., Rîpă M.R., Popescu G., Maștaleru A., Oancea A., Leon M.M. (2025). *Globicatella sanguinis*—A literature review of case reports. Medicina.

[10] Moreland R.B., Choi B.I., Geaman W., Gonzalez C., Hochstedler-Kramer B.R., John J., Kaindl J., Kesav N., Lamichhane J., Lucio L. (2023). Beyond the usual suspects: Emerging uropathogens in the microbiome age. Front. Urol..

[11] Rahmati E., Martin V., Wong D., Sattler F., Petterson J., Ward P., Butler-Wu S.M., She R.C. (2017). *Facklamia* species as an underrecognized pathogen. Open Forum Infect. Dis..

[12] Werner G., Fleige C., Fessler A.T., Timke M., Kostrzewa M., Zischka M., Peters T., Kaspar H., Schwarz S. (2012). Improved identification including MALDI-TOF mass spectrometry analysis of group D streptococci from bovine mastitis and subsequent molecular characterization of corresponding *Enterococcus faecalis* and *Enterococcus faecium* isolates. Vet. Microbiol..

[13] Mahmood H.-A.R., Abdullah B.A. (2025). Classification, importance, and pathogenicity of *Facklamia* species and related bacteria. Int. J. Med. All Body Health Res..

[14] Collins M.D., Lawson P.A., Monasterio R., Falsen E., Sjöden B., Facklam R.R. (1998). *Facklamia ignava* sp. nov., isolated from human clinical specimens. J. Clin. Microbiol..

[15] LaClaire L., Facklam R. (2000). Antimicrobial susceptibilities and clinical sources of *Facklamia* species. Antimicrob. Agents Chemother..

[16] Healy B., Beukenholt R., Tuthill D. (2005). *Facklamia hominis* causing chorioamnionitis and puerperal bacteraemia. J. Infect..

[17] Safavi S., Tufnell M., Bhalla A. (2010). Multi-territory ischaemic strokes and subacute bacterial endocarditis. BMJ Case Rep..

[18] Ananthakrishna R., Shankarappa R.K., Jagadeesan N., Math R.S., Karur S., Nanjappa M.C. (2012). Infective endocarditis: A rare organism in an uncommon setting. Case Rep. Infect. Dis..

[19] Corona P.S., Haddad S., Andrés J., González-López J.J., Amat C., Flores X. (2014). Case report: First report of a prosthetic joint infection caused by *Facklamia hominis*. Diagn. Microbiol. Infect. Dis..

[20] Abat C., Garcia V., Rolain J.-M. (2016). *Facklamia hominis* scapula abscess, Marseille, France. New Microbes New Infect..

[21] Schlipköter M., Grieser T., Forst H. (2017). Ungewöhnliche Komplikation nach Anlage eines Periduralkatheters. Anaesthesist.

[22] Goméz-Luque J.M., Foronda-García-Hidalgo C., Gutiérrez-Fernández J. (2019). Balanopostitis por *Facklamia hominis* en Pediatría [Balanopostitis by *Facklamia hominis* in pediatrics]. Rev. Esp. Quimioter..

[23] Kim T.Y., Jo J., Kim N., Park H., Roh E.Y., Yoon J.H., Shin S. (2019). *Facklamia hominis* isolated from a wound: A case report and review of the literature. Ann. Clin. Microbiol..

[24] Gahl M., Stöckli T., Fahrner R. (2020). *Facklamia hominis* bacteremia after transurethral resection of the prostate: A case report. BMC Urol..

[25] Ait Tamlihat Y., Violette J., Labraousse J., Bregeaud D., Vincent J.F. (2020). Identification of *Facklamia hominis* in a case of stercoral peritonitis: A case report and review of the literature. Infect. Dis. Diagn. Treat..

[26] Basal Y., Oommen J., Faraj U., Acho R., Aravapally A. (2021). *Facklamia hominis* in hidradenitis suppurativa. JAAD Case Rep..

[27] Pérez-Cavazos S., Cisneros-Saldaña D., Espinosa-Villaseñor F., Castillo-Bejarano J.I., Vaquera-Aparicio D.N., Sánchez-Alanís H., Santos A.M.-D.L. (2022). *Facklamia hominis* pyelonephritis in a pediatric patient: First case report and review of the literature. Ann. Clin. Microbiol. Antimicrob..

[28] Agrawal T.L., Chang J.W. (2024). *Facklamia hominis* isolated from infected sebaceous cyst on the breast. Int. Surg. J..

[29] Dharmalingam D., Semalaiyappan J., Thirumal S., Kuttiatt V.S. (2025). Unveiling Facklamia: Detection of an emerging microbe in the skin microbiome of patients with filarial lymphedema. Front. Cell. Infect. Microbiol..

[30] Joyce C., Halverson T., Gonzalez C., Brubaker L., Wolfe A.J. (2022). The urobiomes of adult women with various lower urinary tract symptoms status differ: A re-analysis. Front. Cell Infect. Microbiol..

